# Applicability of pan-TRK immunohistochemistry for identification of *NTRK* fusions in lung carcinoma

**DOI:** 10.1038/s41598-021-89373-3

**Published:** 2021-05-07

**Authors:** Simon Strohmeier, Iva Brcic, Helmut Popper, Bernadette Liegl-Atzwanger, Jörg Lindenmann, Luka Brcic

**Affiliations:** 1 Diagnostic and Research Institute of Pathology, Medical University of Graz, Neue Stiftingtalstrasse 6, 8010 Graz, Austria; 2 Division of Thoracic Surgery and Hyperbaric Surgery, Department of Surgery, Medical University of Graz, Auenbruggerplatz 29/3, 8036 Graz, Austria

**Keywords:** Cancer, Molecular biology, Biomarkers, Molecular medicine

## Abstract

In the last two decades, various therapies have been introduced for lung carcinoma patients, including tyrosine-kinase inhibitors for different mutations. While some of them are specific to specific tumor types, others, like *NTRK1–3* fusions, are found in various solid tumors. The occurrence of an *NTRK1,2 or 3* fusion acts as a biomarker for efficient treatment with NTRK inhibitors, irrespectively of the tumor type. However, the occurrence of the *NTRK1–3* fusions in lung carcinomas is extremely rare. We performed a retrospective analysis to evaluate the applicability of immunohistochemistry with the pan-TRK antibody in the detection of *NTRK* fusions in lung carcinomas. The study cohort included 176 adenocarcinomas (AC), 161 squamous cell carcinomas (SCC), 31 large-cell neuroendocrine carcinomas (LCNEC), and 19 small cell lung carcinomas (SCLC). Immunohistochemistry (IHC) was performed using the pan-TRK antibody (clone EPR17341, Ventana) on tissue microarrays, while confirmation for all positive cases was done using RNA-based Archer FusionPlex MUG Lung Panel. On IHC staining, 12/387 samples (3.1%) demonstrated a positive reaction. Ten SCC cases (10/161, 6.2%), and two LCNEC cases (2/31, 6.5%) were positive. Positive cases demonstrated heterogeneous staining of tumor cells, mostly membranous with some cytoplasmic and in one case nuclear pattern. RNA-based sequencing did not demonstrate any *NTRK1–3* fusion in our patients’ collective. Our study demonstrates that pan-TRK expression in lung carcinoma is very low across different histologic types. *NTRK1–3* fusions using an RNA-based sequencing approached could not be detected. This stresses the importance of confirmation of immunohistochemistry results by molecular methods.

## Introduction

Lung cancer is one of the most common malignancies worldwide and the leading cause of cancer-related death^1^. However, according to the recent literature, as a result of anti-tobacco campaigns, screening and new therapy options, the mortality in the USA has decreased both in men and women, by 48% and 23%, respectively^2^. In the last couple of years, the real “game-changer” in the therapy of lung carcinoma is immunotherapy. Nevertheless, since the first tyrosine-kinase inhibitors against activating EGFR mutations were introduced a little bit more than a decade ago, there has been an improvement in understanding molecular mechanisms responsible for lung cancer development including the detection of targetable mutations. Although these targetable mutations are present in a small proportion of all lung carcinomas, the number of druggable mutations increases every year. According to the latest international recommendations from 2018^3^, preferred way of predictive testing for advanced non-squamous non-small cell lung carcinomas (NSCLC) is multigene testing including *EGFR, ALK, ROS1, BRAF, MET, HER2, KRAS,* and *RET*. Very recently, Food and Drug Administration (FDA) and European Medicine Agency (EMA) have approved treatment for tumors harboring neurotrophic tyrosine receptor kinase (*NTRK*) gene fusions, as a first-line or subsequent-line of therapy^4^.

Three *NTRK* genes (*NTRK 1, NTRK 2,* and *NTRK 3*) encode the three transmembrane neurotrophin receptors TrkA (NTRK1), TrkB (NTRK2), and TrkC (NTRK3)^5^. TRK receptors play a significant role in the development and functioning of the central and peripheral nervous system^6–8^. However, chromosomal rearrangements of these genes with different partners may cause activation and/or overexpression of TRK receptors resulting in tumor development^9, 10^. *NTRK* fusions are characteristically found in several rare tumors, like congenital infantile fibrosarcoma, congenital “cellular” mesoblastic nephroma, secretory breast carcinoma, or mammary analogue secretory carcinoma of the salivary glands^11–18^. The most common fusion found in about 90% of these cases is *ETV6-NTRK3*^11–18^*.* Unfrequently, *NTRK* fusions are described in other rare tumors, like soft tissue neoplasms, but also in common solid tumors, like NSCLC, colorectal carcinoma, gastrointestinal stromal tumors, papillary thyroid carcinoma, glioma, malignant melanoma, and pancreatic adenocarcinoma^4, 9, 16, 19–27^. Overall incidence of *NTRK* fusions in all solid tumors is very low, accounting for less than 1%. According to the published data, *NTRK* fusions in NSCLC are found in 0.1–1% of cases^8, 28, 29^. However, although rare, targeted therapy induces a response in the vast majority of patients harboring these fusions, and their identification is crucial for further treatment^4, 20, 30, 31^.

The most reliable method to identify *NTRK* fusions is RNA-based massive parallel sequencing (MPS). However, it is not available in every institution, it is time-consuming and expensive. Immunohistochemistry, using a pan-TRK antibody, is an affordable and easily available technique in most pathology laboratories. Therefore, this method has been suggested as an optimal screening tool for a TRK fusion protein expression, which if positive should be confirmed with MPS^32, 33^. Nevertheless, the staining pattern is not uniform and there is no standardized approach for scoring and interpretation of IHC expression^23, 33–35^.

To evaluate the patterns of staining and the applicability of immunohistochemistry with the pan-TRK antibody in the detection of *NTRK* fusions, we performed a retrospective analysis on a lung carcinoma cohort including different tumor subtypes and tested all positive samples with MPS.

## Material and methods

### Study cohort

From the archives of the Diagnostic and Research Institute of Pathology, 387 lung carcinoma cases diagnosed between 1993 and 2012 were selected for this retrospective analysis. All cases were re-evaluated according to WHO 2015 criteria^36^ to confirm a diagnosis and to select adequate tissue areas for the tissue microarray (TMA) construction. Furthermore, all cases were re-staged according to the UICC/AJCC staging from 2017^37^. At the time of the study all patients have passed away, therefore we were not able to obtain informed consents. This study conforms to the principles outlined in the Declaration of Helsinki and was approved by the Ethics Committee of the Medical University of Graz (24-135 ex11/12).

The cohort included 176 adenocarcinomas (AC), 161 squamous cell carcinomas (SCC), 31 large-cell neuroendocrine carcinomas (LCNEC), and 19 small cell lung carcinomas (SCLC). All patients underwent surgery and the resection material was used for further analysis. Clinicopathological data are summarized in Table 1. When looking at individual cancer subtypes, in AC median age was 64 years (range 41–84). The majority of the patients in this groups were males (110/176, 62.5%), and according to UICC/AJCC 80/176 (45.5%) were in stage I, 63/176 (35.8%) in stage II, 29/176 (16.5%) in stage III and 3/176 (1.7%) in stage IV. The remaining single case could not be staged due to a lack of data. The median age in SCC was 65 years (range 41–89). The vast majority of patients with SCC were male (141/161, 87.6%). 52/161 (32.3%) were stage I carcinomas, 82/161 (50.9%) were stage II, 23/161 (14.3%) stage III, and 1 (0.6%) stage IV. For three patients data were not available for further staging. In the LCNEC patients’ group, the median age was 64 (range 37–89), 19/31 (61.3%) patients were male. According to UICC/AJCC classification, 7/31 cases (22.6%) were stage I, 13 (41.9%) stage II, 7/31 (22.6%) stage III. For 4 cases we were not able to determine the stage due to a lack of data. The median age in the SCLC group was 65 years (range 52–86), with male predominance (15/19, 78.9%). The majority of patients (12/19, 63.2%) were in stage II, 5/19 (26.3%) were stage III and 2 (10.5%) were stage IV.

**Table 1:** Study cohort with the results of immunohistochemistry with a pan-TRK antibody.

	n	%
**Histology**		
Adenocarcinoma	176	45.5
Squamous cell carcinoma	161	41.6
Large-cell neuroendocrine carcinoma	31	8.0
Small cell lung carcinoma	19	4.9
**Age at diagnosis**		
Median	64	
Range	37–89	
**Gender**		
Male	285	73.6
Female	102	26.4
**Stage at diagnosis (UICC 2017)**		
I	139	35.9
II	170	43.9
III	64	16.5
IV	6	1.6
Undefined (lack of data)	8	2.1
**NTRK positive**	12	3.1
Adenocarcinoma	0/176	0
Squamous cell carcinoma	10/161	6.2^a^
Large-cell neuroendocrine carcinoma	2/31	6.5^a^
Small cell lung carcinoma	0/19	0

^a^% of the number of each histologic subtype, not of the whole study cohort.

### Immunohistochemical analysis

For TMA construction, four 0.6 mm cores were used from each tumor sample, which was formalin-fixed and paraffin-embedded (FFPE), using TMA Grand Master (3DHistech, Budapest, Hungary). For immunohistochemical (IHC) analysis, 4 µm-thick TMA sections were used. Pan-TRK immunohistochemical staining (rabbit monoclonal antibody, clone EPR17341, RTU, Roche, Ventana) was performed on the Benchmark Ultra using iVIEW DAB Detection Kit (both from Ventana Medical Systems, Tucson, AZ). As the positive controls, normal appendix and brain samples were used. Furthermore, one tumor with NGS-proven NTRK fusion was used as additional control of the staining, in this tumor, 80% of tumor cells showed cytoplasmic positivity. The evaluation of staining included a percentage of positive tumor cells, intensity of staining (weak, moderate, strong), and localization of staining (cytoplasmic, membranous, nuclear). Any staining stronger than a background in ≥ 1% of tumor cells, regardless of localization, was regarded as positive^23^. The evaluation was performed by three authors (IB, LB, SS) and was expressed as a mean value of all cores available for analysis per patient. Any discrepancies were resolved by joint discussion. Whole sections of positive cases were also stained with pan-TRK antibody to investigate the presence of intratumoral heterogeneity.

### Molecular analysis

All cases with positive IHC reactions were sent for further analysis using RNA-based Archer FusionPlex MUG Lung Panel (ArcherDX, Boulder, CO). RNA was isolated from the 5–8, 10 μm thick, FFPE sections cut from a representative block using macrodissection and the Maxwell RSC RNA FFPE kit. RNA quantification was performed using ribogreen fluorescence, and 250 ng total RNA was used. NGS libraries were sequenced on Ion S5 (Ion Torrent, Thermo Fischer, Waltham, MA) using the Ion PI Hi-Q Sequencing 200 kit (Thermo Fischer, Waltham, MA). ArcherDX Analysis software Version 5.1.3. (ArcherDX, Boulder, CO) was used for data analysis.

### Ethics approval

The study was approved by the Ethics Committee of the Medical University of Graz (24-135 ex11/12), which granted the waiver for the informed consent for this specific study, since, unfortunately, at the time of this study all patients whose samples were used have already passed away.

## Results

### Immunohistochemical analysis

**Figure 1:**
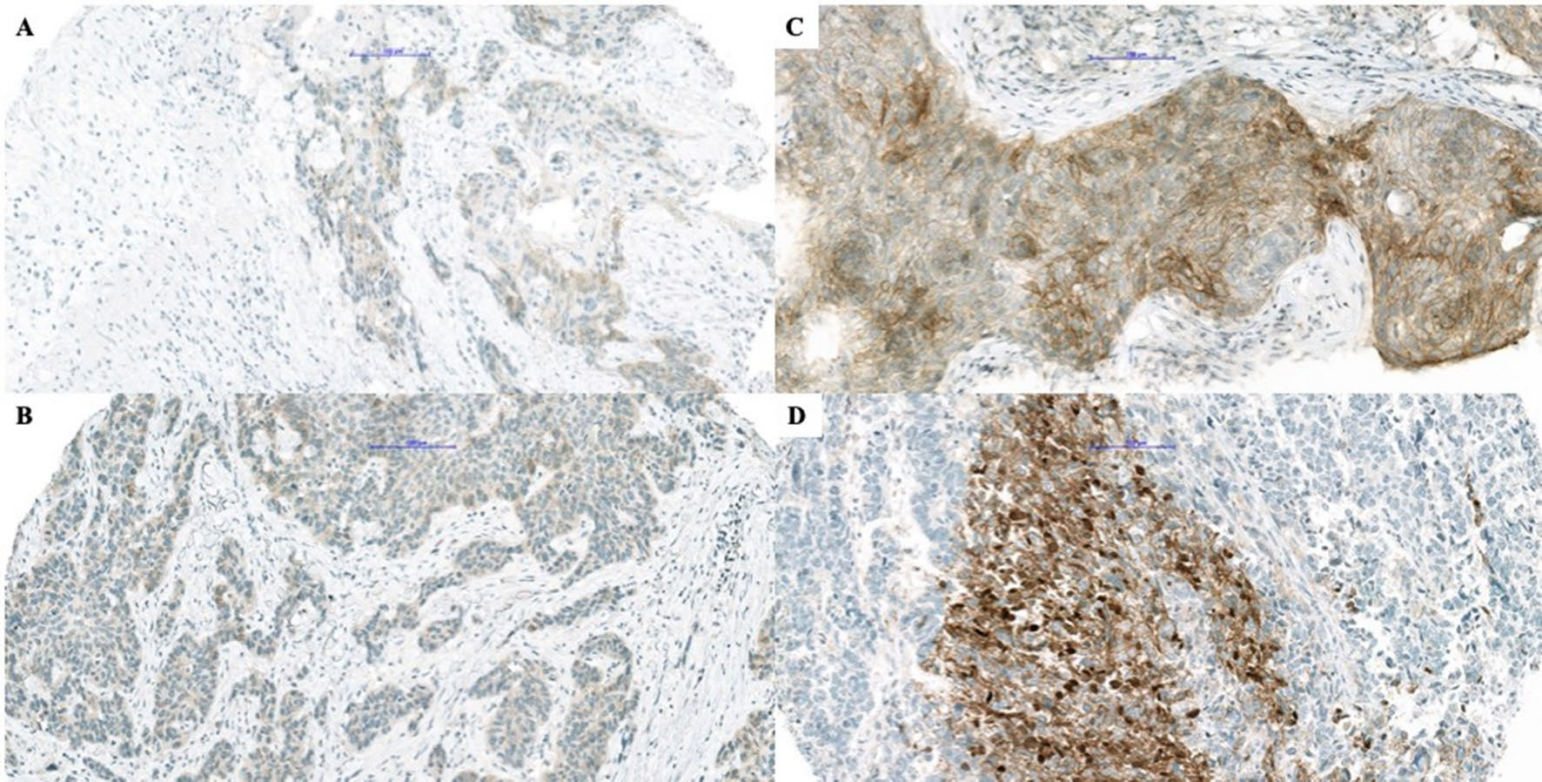
Presentation of positive immunohistochemical reactions. Different patterns of immunohistochemical staining with pan-Trk antibody in squamous cell carcinoma **(A–C)** and large cell neuroendocrine carcinoma **(D)**. In (**A,B)** there is a weak cytoplasmic reaction, while one can appreciate a strong membranous reaction in **(C)**, and nuclear and membranous reaction in **(D)** (bar = 100 µm).

On IHC staining, 12/387 samples (3.1%) demonstrated positive reaction, including ten SCC cases (10/161, 6.2%), and two cases in the LCNEC group (2/31, 6.5%). 8/10 SCC (80%) showed weak cytoplasmic and 2/10 (20%) strong membranous staining pattern, with no more than 10% of positive tumor cells in all but one SCC which demonstrated strong membranous staining in 70% of tumor cells (Fig. 1). Strong pan-TRK expression was found in one (50%) LCNEC with cytoplasmic staining in 60% of tumor cells. The second positive LCNEC case presented strong cytoplasmic and focal nuclear positivity in 25% of tumor cells (Fig. 1). None of the analyzed AC and SCLC showed a positive IHC reaction.

Furthermore, the stained whole sections of all positive tumor samples demonstrated clear heterogeneity in the staining pattern.

### Molecular analysis

In seven cases no *NTRK1–3* fusions could be proven. Five cases could not be evaluated due to insufficient RNA quality, despite repeated analysis using different tumor tissue blocks. Interestingly, the age of the blocks did not play a role in this rather high failure rate.

## Discussion

Results of our study analyzing pan-TRK expression in lung carcinoma demonstrate very low positivity across different histologic types, without any confirmed *NTRK1–3* fusions using an RNA-based sequencing method. This stresses the importance of confirmation of immunohistochemistry results by molecular methods.

Our results are in concordance with recent studies that also clearly demonstrated a very low occurrence of these mutations in lung carcinoma. In one of the first studies looking at the oncogenic and drug-sensitive *NTRK* rearrangements in the lung AC, Vaishnavi et al*.* have found *NTRK1* fusions in 3/91 patients with lung AC (Table 2)^29^. Such a high incidence (3.3%) was very promising, however, later studies were not able to confirm this finding. Of note, this study had a selection bias, since only tumors without any other already known oncogenic driver mutations were included. In 2018, Gatalica et al*.* presented results of 11,502 solid tumors´ samples submitted for molecular profiling, using RNA-based ArcherDx FusionPlex Assay for fusion detection. Among other tumor types, 4,073 NSCLC were included, and in 4 cases *NTRK1–3* fusions (0.10%) were found (see Table 2)^38^. A multicentric study in 2018 by Farago et al*.* found 11 NSCLC (0.23%) harboring *NTRK1* and *NTRK3* fusions (listed in Table 2) in 4872 screened cases, using also RNA-based MPS^10^. The majority of positive cases were AC (9), with one SCC and one LCNEC. Very recently, the largest cohort of RNA-based *NTRK1–3* fusion analysis was performed on 38,095 solid tumor samples, including 3,993 lung AC^13^. Interestingly, they found the same incidence of 0.23% of *NTRK* fusion-positive AC (9/3,993). In summary, all these studies together included 27 lung carcinomas harboring *NTRK1–3* fusions, 25 being AC^10, 13, 29, 38^. Among these, the *NTRK1* gene was the most common fusion partner (17/25, 68%), followed by *NTRK3* (6/25, 24%), and only rarely *NTRK2*. One published SCC and one LCNEC harbored an *NTRK3* gene fusion^10^.

**Table 2:** Distribution of fusion partners according to the histologic type in published studies.

Study	Histologic type	Number of analyzed lung carcinoma	Positive cases (histologic type)	Percentage of positive cases	Detected fusions
Vaishnavi et al., 2013	AC	91	3 (AC)	3.3%	*CD74-NTRK1* *MPRIP-NTRK1* *NTRK1*^a^
Gatalica et al., 2018	NSCLC	4073	4 (AC)	0.10%	*TPM3-NTRK1* *SQSTM1-NTRK2* *ETV6-NTRK3* *ETV6-NTRK3*
Farago et al. 2018	NSCLC	4872	9 (AC)	0.23%	*IRF2BP2-NTRK1* *IRF2BP2-NTRK1* *MPRIP-NTRK1* *SQSTM1-NTRK1* *SQSTM1-NTRK1* *TPM3-NTRK1* *TPR-NTRK1* *ETV6-NTRK3* *ETV6-NTRK3*
			1 (SCC)		*ETV6-NTRK3*
			1 (NE)		*SQSTM1-NTRK3*
Solomon et al., 2020	AC	3993	9 (AC)	0.23%	*EPS15-NTRK1* *EPS15-NTRK1* *F11-NTRK1* *IRF2BP2-NTRK1* *TFG-NTRK1* *TPM3-NTRK1* *STRN-NTRK2* *RBPMS-NTRK3* *SQSTM1-NTRK3*

*AC* adenocarcinoma, *NSCLC* non-small cell lung carcinoma, *SCC* squamous cell carcinoma, *NE* neuroendocrine carcinoma.

^a^This fusion was detected using a break-apart FISH probe detecting different *NTRK1* fusions.

Current recommendations suggest that RNA-based MPS technologies are the golden standard to detect *NTRK* gene fusions in all solid tumors^33, 39^. However, RNA-based MPS methods are not available in all pathology laboratories, are very expensive and time-consuming. Therefore, immunohistochemistry is used as a screening method to search for pan-TRK protein expression that may be caused by *NTRK*-fusions. In contrast to MPS immunohistochemistry is widely available, does not require as much tumor tissue as molecular methods, is fast and cheap. Currently, there is no consensus about the best anti-NTRK antibody to be used. There are monoclonal antibodies detecting specific proteins, for example, rabbit TrkA (clone ab76291, Abcam), rabbit TrkB (clone J9.777.7 Thermo Fisher or clone EPR 17805-146 from Abcam), or the ones detecting overexpression of all NTRK1–3 proteins (rabbit pan-TRK antibody, clone EPR17341 from Roche/Ventana or Abcam) and A7H6R (Cell Signaling). As far as we know, there is only one report comparing two different clones, EPR17341 (both from Ventana and Abcam) and A7H6R (Cell Signaling), demonstrating comparable performance in different laboratories^40^. Both previously mentioned studies (from Gatalica et al*.* and Solomon et al*.*) used clone EPR17341 from Abcam. The first study by Gatalica showed an overall sensitivity of 75%, with 95.9% specificity^38^. Solomon et al*.* demonstrated lower specificity (81.1%) and nicely showed that sensitivity is not the same for *NTRK1*, *NTRK2,* and *NTRK3* gene fusions, being 96.2%, 100%, and 79.4% respectively^13^. These results are in contrast to other published studies where the sensitivity of 95.2% and 97%, and very high specificity, 100%, and 98%, respectively were found^23, 34^. An additional study using also EPR17341 from Abcam demonstrated high sensitivity, but lower specificity^35^. All mentioned studies have demonstrated that IHC is much better at the detection of *NTRK1* and *NTRK2* gene fusions, but lacks sensitivity for the detection of *NTRK3* gene fusions. What is the reason for NTRK1–3 expression, other than fusion in *NTRK1–3* genes, is not completely clear. An explanation is probably in genetic and/or epigenetic changes, like activating mutations which are found in some lung neuroendocrine carcinomas^41^. When we combine these facts with the previously mentioned incidence of *NTRK1–3* gene fusions in lung carcinoma, it is more than possible that a certain proportion of samples harboring *NTRK* fusions (especially *NTRK3*) are being missed using IHC as a screening method. This is a crucial point to have in mind when deciding which method of testing to use.

For this study, we have used pan-TRK ready-to-use assay (clone EPR17341 Roche/Ventana) and analyzed protein expression not only in lung AC but also in SCC, LCNEC, and SCLC. Overall, we have found 12 positive samples out of 387 cases (3.1%). Interestingly, 6.5% of analyzed LCNEC were positive, as well as 6.2% of SCC. Using an RNA-based MPS approach, no *NTRK1–3* fusions were detected.

In a very recent study, Leal et al. used a cocktail of pan-TRK (clone A7H6R, Cell Signaling), ALK, and ROS-1 antibodies on TMA and found a positive reaction in 0.4% of NSCLC (2/522), while all SCLC (105) were negative for this antibody cocktail. After RNA sequencing, two positive NSCLC demonstrated ALK fusions. In this study, no NSCLC or SCLC with *NTRK1–3* gene fusions has been detected as well^42^. Using the same antibody as in our study, with some protocol modifications, Elfving et al*.* evaluated the expression of pan-TRK in 617 NSCLC^43^. They have found a weak positive reaction in 17 cases (2.8%) and in an additional 1.8% of cases (11/617) moderate to strong staining was observed. The majority of IHC positive cases, like in our study, were SCC. They have also found the staining pattern to be heterogeneous, and rarely more than 80% of tumor cells showed positive staining. This study is similar to ours concerning the case selection, since both studies used surgical material to construct TMAs, and the majority of patients included in the study were in lower clinical stages, in comparison to other published data. Analogous to our study, none of the cases demonstrated *NTRK1–3* gene fusion using RNA-based sequencing.

Our study has several limitations. First, it is a single-center, retrospective study, using older FFPE tissue. This can theoretically influence IHC results, or like in some of our cases make RNA-based molecular analysis impossible, although the average age of our blocks did not differ between successfully tested and failed samples. However, the influence of preanalytical variables can here not be excluded. Altogether, the number of cases was not very high, especially for some histologic groups (LCNEC and SCLC), which might explain the rather high incidence of IHC positive cases in SCC, LCNEC, and SCLC groups. Moreover, the IHC analysis was performed using a TMA-based approach, which, although 4 cores from each tumor were used, cannot compensate for the staining heterogeneity of tumors. This, on the other hand, represents the real-life situation where we are dealing with small biopsies in a large number of patients. Lastly, all tumors included in this study were not analyzed using RNA-sequencing and the rate of the IHC false-negative cases could not be evaluated.

Our study has confirmed that protein expression does not imply the presence of *NTRK1–3* gene fusions and has, therefore, to be verified, ideally by RNA-based MPS. Furthermore, *NTRK1–3* fusions occur infrequently in lung carcinomas. However, whether the protein expression is also important for the therapeutic effect, even without fusion, and the real number of cases harboring these rare fusions that we miss using immunohistochemistry as a screening should be clarified in further studies.

## Data Availability

Available upon reasonable request.
